# Solar-Driven Photocatalytic Degradation of Dye Pollutant Using MnO_2_-Modified Biochar via Fenton-like Reactions

**DOI:** 10.3390/polym18091119

**Published:** 2026-04-30

**Authors:** Jorge A. Soto Sandoval, Abdullah Al Ragib, Janusz Kozinski, Sudip K. Rakshit, Kang Kang

**Affiliations:** 1 Biorefining Research Institute (BRI) and Department of Chemical Engineering, Lakehead University, Thunder Bay, ON P7B 5E1, Canada; jasando1@lakeheadu.ca (J.A.S.S.); aragib@lakeheadu.ca (A.A.R.); jkozinsk@lakeheadu.ca (J.K.); srakshit@lakeheadu.ca (S.K.R.); 2 Escuela de Ingeniería y Ciencias, Tecnologico de Monterrey, Ave. Eugenio Garza Sada 2501, Monterrey 64700, Mexico

**Keywords:** biochar catalysts, solar-driven catalysis, Fenton-like reactions, environmental remediation, waste valorization, functional carbon materials

## Abstract

Manganese dioxide (MnO_2_) modified biochar catalysts derived from biomass and waste polymer feedstocks were synthesized and evaluated as heterogeneous Fenton-like catalysts for solar-driven degradation of Rhodamine B (RhB) in aqueous systems. Biochars produced from maple wood and plastic waste (high-density polyethylene) provided porous carbon matrices with oxygen-rich surface functionalities that enabled effective MnO_2_ loading and catalytic activity. Photocatalytic experiments conducted under real sunlight using a solar-collector reactor demonstrated faster RhB degradation compared to a conventional ultraviolet (UV) system, confirming the advantage of solar-driven operation. Complete RhB removal was achieved at initial concentrations of 100–300 ppm, whereas higher dye concentrations (500 ppm) exceeded the catalytic capacity within the tested reaction time. Kinetic analysis revealed catalyst-dependent reaction behaviors, indicating that degradation pathways were strongly influenced by the biopolymer-derived carbon structure and MnO_2_ dispersion. Degradation efficiency was correlated with solar irradiance and reactor temperature, with higher UV index conditions enhancing catalytic performance. Reusability tests showed that the catalysts remained active over multiple cycles, although gradual decreases in reaction rates and catalyst recovery were observed. These results demonstrate the potential of biopolymer-derived carbon materials as effective solar-driven catalysts for wastewater treatment applications.

## 1. Introduction

Intensive industrial growth and rapid urban expansion have contributed significantly to producing dye-containing wastewater, imposing serious environmental impacts [1]. Among different synthetic dyes, Rhodamine B (RhB) is one of the most toxic coloring agents in textile industries, and its highly stable, water-soluble, complex structure and non-biodegradable nature mean that it persists within the environment. This synthetic dye is known for being highly carcinogenic and showing mutagenic effects on human and animal development, including on the skin, eyes, and gastrointestinal, respiratory, and nervous systems [2]. Due to its complicated molecular structure, RhB also demonstrates significant resistance to microbial degradation processes, as well as solar-induced photolytic breakdown [3].

Traditional dye treatment processes like sedimentation, filtration, coagulation, aeration, and chemical flocculation have been proven to be minimally effective for the removal of RhB dye. However, there are drawbacks to these mechanisms: the need to set up vast treatment plants, high energy consumption, production of hazardous compounds, and bad odor as a result of the implementation of dye removal techniques [4]. Although the adsorption process of sediment and soil particles has been thought to be a transient sink for RhB dye, it is unable to achieve complete degradation. Rather, under favorable conditions, it might facilitate the re-introduction of RhB into the aquatic environment [3]. Biological treatment such as biofilm and activated sludge also have several shortcomings including expensive operating systems, prolonged pre-preparation cycles and the swelling of sludge [5].

Advanced Oxidation Processes (AOPs) have recently been proven to be one of the most promising treatment methods for removing organic contaminants from wastewater. Several advantages of AOPs have been reported, such as complete mineralization capacity in harmless byproducts and a rapid rate of oxidation reaction [6]. The main feature of the Fenton process, Fenton-like processes, photo-Fenton, ultrasound-based, and electrochemical Fenton AOPs is the generation of extremely reactive oxygen species (ROS) like hydroxyl radicals (•OH), superoxide radicals (•O_2_^−^) and sulfate radicals (SO_4_•^−^) that essentially degrade wastewater organic contaminants and mineralize them into non-toxic substances like CO_2_, water, and salts [7]. The major drawbacks of using homogenous conventional AOPs are the narrow pH range of 2–4 for the Fenton process, and high energy and electricity requirements for sono-based and electrochemical AOPs, respectively. UV-based photochemical AOPs show lower quantum yields, and potentially harmful byproducts are generated in the case of ozone-oxidation AOPs [8].

Metal-oxide-based heterogeneous Fenton-like AOPs have attracted much attraction as highly promising and efficient techniques due to their wider range of operational pH and minimum sludge production [9]. Manganese dioxide (MnO_2_) has been identified as a potentially efficient heterogeneous Fenton-like catalyst because of its exclusive physiochemical characteristics like redox flexibility, enhanced surface area with different oxidation states and negligible ecotoxicological effects [10]. However, MnO_2_ has an augmented surface reactivity due to its intense *Van der Waals* interactions that contribute to aggregation. Therefore, the active surface area becomes reduced, which lowers the catalytic activity significantly [11]. Biochar is typically produced through the thermal breakdown of carbon-rich biomass under oxygen-limited conditions. Over the past few years, biochar has received considerable attention as a cost-effective promising adsorbent capable of removing harmful organic and inorganic compounds from the contaminated wastewater [12]. Other than being an effective adsorbent, biochar also possesses redox potential and electron shuttling capability that make it a highly efficient catalyst for reactive species, including •OH radical generation via hydrogen peroxide (H_2_O_2_) activation [13]. Biochar acts as a sustainable support material for the synthesis of a heterogeneous Fenton-like process catalyst that can efficiently inhibit the agglomeration tendency of MnO_2_, thus ensuring its maximum utilization. Therefore, biochar-supported Mn-loaded catalysts have high potential to be efficiently utilized in heterogeneous Fenton-like AOPs for environmental remediation.

The solar-driven photo-Fenton process has become a novel approach nowadays, as solar radiation is a sustainable, ubiquitous and renewable energy source [14]. The low energy requirement of the solar Fenton process provides a significant advantage over UV-assisted or other conventional approaches due to significantly reduced electricity consumption [15]. In addition, the solar-driven photo-Fenton process enhances the oxidative capacity of the whole system by generating additional oxidative radicals through the redox cycle and H_2_O_2_ breakdown under solar irradiation. However, despite being a promising technology, photo-Fenton processes in most existing studies are still confined to the use of solar simulators rather than real sunlight. As such, the dye degradation capacity of photocatalysts under the wide spectrum of natural sunlight, the effective contribution of both visible light and UV light of natural solar irradiation to photocatalyst activity, and actual performance and scalability under real sunlight operation are not explicitly explored. In addition, most of the existing studies demonstrated simple kinetic models such as *Langmuir–Hinshelwood* models and pseudo-first- or second-order kinetics without appropriate justification for how the kinetics are modeled for different types of catalysts [16]. Both light energy and temperature play crucial roles in photo-Fenton reactions. However, most wastewater treatment studies have shown the independent influences of temperature and energy on the degradation capacity of photocatalysts in lab set ups [17]. Therefore, a research gap remains with regard to how the lighting intensity of solar energy and reactor temperature jointly influence the degradation kinetics of different types of photocatalysts.

In this study, MnO_2_-modified biochar catalysts developed using woody biomass (maple wood) and waste feedstocks, aerobic paper sludge (APS) and high-density polyethylene (HDPE) were systematically characterized and evaluated for solar-driven RhB degradation in water. Moreover, compared to previously reported MnO_2_/biochar Fenton-like systems, this work also provides a comprehensive assessment of degradation efficiency and catalyst performance, with a special focus on the reaction kinetics under real and variant solar irradiation conditions. Specifically, the other novelty of this work lies not only in the integration of real full-spectrum sunlight, catalyst-specific kinetic modeling across catalysts derived from diverse biomass sources, and energy balance-based reactor efficiency evaluation, but also in their systematic coupling under realistic outdoor conditions. This study elucidates a systematic and reliable performance prediction and facilitates the scalable implementation of a sustainable photo-Fenton-like system.

## 2. Materials and Methods

### 2.1. Materials

Maple wood (MW) chips, aerobic paper sludge (APS), and high-density polyethylene (HDPE) were procured from local suppliers in Thunder Bay, ON, Canada. Manganese (IV) dioxide (MnO_2_) and Rhodamine B (RhB) were purchased from Sigma-Aldrich (Oakville, ON, Canada). Deionized (DI) water was obtained from Nanopure Water, Barnstead (Waltham, MA, USA). The compressed N_2_ gas used for the catalyst preparation was purchased from Linde Canada Inc. (Mississauga, ON, Canada).

### 2.2. Biochar Preparation

In this study, to prepare the Mn-loaded Fenton catalysts, we developed a two-step process: Step 1: biochar production using biomass and other waste feedstock; and Step 2: Mn-based Fenton catalyst production using biochar produced in Step 1 and MnO_2_.

In Step 1, for biochar production, three different types of biomass were chosen: maple wood (MW), aerobic paper sludge (APS), and high-density polyethylene (HDPE). All biomass samples were oven-dried at 105 °C for 12 h. Then, the co-pyrolysis/pyrolysis process was conducted using an Across International tube furnace STF1200-60X1000 (Sparks, NV, USA). MW–HDPE and MW–APS feedstocks were prepared at a 1:1 mass ratio.

The pyrolysis process was conducted in an inert nitrogen flow of 250 mL/min, at 700 °C and 2 h of reaction with a heating rate of 10 °C/min. In Step 2, MnO_2_ in a mass ratio of MnO_2_:biochar = 1:3 was loaded onto the biochar via co-pyrolysis; each sample was adjusted to the total initial weight of 3 g. The reaction was conducted in an inert environment with a 250 mL/min flow rate of N_2_, 800 °C, and 2 h of reaction. In this study, three Fenton catalysts were prepared and designated as MW–Mn, MW–HDPE–Mn, and MW–APS–Mn.

### 2.3. Biochar Catalyst Characterization Techniques

X-ray diffraction (XRD) analysis was performed to determine crystallographic structure using a PANalytical X’pert Pro diffractometer (Almelo, The Netherlands) with Cu Kα radiation within 6–85° at a scanning rate of 2°/min. Fourier transform infrared (FTIR) was used to identify surface functional groups on biochar catalysts using a Bruker Tensor 37 spectrophotometer equipped with Diamond ATR accessory (Billerica, MA, USA). Scanning electron microscopy (SEM) using a Hitachi SU-70 (Hitachinaka, Japan) and energy-dispersive spectroscopy (EDS) were performed to examine surface morphology and localized elemental distribution.

### 2.4. Fenton Reaction Sample Preparation

RhB was selected as the target dye due to its well-documented adverse effects on both human health and the environment. Moreover, its molecular structure—characterized by strong covalent bonds within the aromatic core and external functional groups—renders it highly resistant to degradation. Consequently, the breakdown of RhB remains a critical challenge in water treatment research and industrial applications. The sample of RhB-polluted water was prepared in different concentrations with distilled water (i.e., 100, 300, and 500 ppm), and the pH was adjusted to 2.7 using HCl at 1 M.

To prepare the reaction, 30 mL of the RhB solution was measured, followed by the addition of 10 mg of biochar, which was shaken for 3 min at 330 RPM in a platform shaker. Finally, 150 uL of H_2_O_2_ was added to the solution to initiate the reaction.

### 2.5. Reaction Set-Up and Analytical Calculations

#### 2.5.1. Solar Fenton Reactor

A commercial GoSun portable solar oven (Cincinnati, OH, USA) was used as the reactor for all solar Fenton experiments. All experiments under natural sunlight conditions were performed in Thunder Bay, ON, Canada (48°25′06.3″ N 89°15′31.4″ W) between 30 June and 12 September 2025.

For each run, the reactor was placed on a flat ground surface facing the sun, and recalibrated every 15 min to track the sunlight. The measurement of the surface temperature of the reactor surface was performed using a Proster dual-channel thermocouple (Shenzhen, China). The reaction sample mixture was prepared in a 50 mL transparent glass tube (used as the reaction vessel), placed in a silicone baking pan above the tray of the reactor. Each mixture was sampled every 5 min in a 30, 45, or 60 min experiment. All experiments were performed between 11 am and 2 pm, ensuring the optimum sunlight conditions with fewer clouds and the highest UV index during the day.

#### 2.5.2. Dye Degradation Efficiency Evaluation and Reaction Kinetics Analysis

For RhB concentration determination, the *Beer–Lambert* law relationship between absorbance and concentration was used, expressed in Equation (1):

(1) A=εlC

where A is the absorbance (measured using a Varian 50 Bio UV-visible spectrophotometer (Markham, ON, Canada) between 700 and 400 nm); ε is the molar extinction coefficient, a constant that depends on the substance and the spectrophotometer; *l* is the path length that depends on the width of the cuvettes where light is passing through; and C is the substance concentration in the solution. The molar extinction coefficient was determined with Equation (1) solved for ε, and to do so, we measured the absorbance of the RhB solution before any light exposition, but because of its common high absorbance value (>1), a dilution factor was needed so that all measurements could be observed in an absorbance range from 0 to 1; for 100 ppm and 300 ppm experiments the dilution factor was 1:30, and for 500 ppm experiments the dilution factor was 1:60. For the dye degradation results, data absorbance vs wavelength was plotted using Origin Pro version 2024, setting the wavelength with higher absorbance of the initial RhB solution and collecting the absorbance value of each sample with the Data Highlighter tool included in version 2024. The concentration of RhB at each experimental stage was calculated using the *Beer–Lambert* law rearranged to solve for concentration.

To analyze the reaction kinetics, the order of the reaction was determined using the equations of the Integral Method of Data Analysis (IMDA); this method is a linear adjustment for the data, and the closer the R^2^ value is to 1, the more precisely the reaction behaves as zero-, first-, or second-order. Equation (2) describes a zero-order reaction, Equation (3) describes a first-order reaction and Equation (4) describes a second-order reaction, respectively.

(2) $$C_{A}=C_{A0}-kt$$

(3) $$\ln\left(\frac{C_{A0}}{C_{A}}\right)=kt$$

(4) $$\frac{1}{C_{A}}-\frac{1}{C_{A0}}=kt$$

In the above equations, $C_{A}$ is the concentration that the sample has at that time of reaction; $C_{A0}$ is the initial concentration of the experiment, which was previously known while preparing the dye solution; k is the kinetic constant of the reaction, which describes how fast the reaction is behaving and can be calculated using the lineal tendency equation; and *t* is the time in min when the sample was taken.

#### 2.5.3. UV Reactor

To compare the efficiency of solar Fenton systems and normally reported UV Fenton systems, we also conducted some controlled experiments using a lab-scale UV reactor (Dongguan, China). For the UV reactor system, a 365 nm UV lamp was used and placed inside an aluminum cover to ensure an enclosed environment at room temperature. The reaction’s solution was placed in a polypropylene centrifuge tube, collecting samples every 5 min for 30 min.

#### 2.5.4. Irradiation Efficiency Calculation Based on UV Index

According to the World Health Organization, the UV index is a simple, standardized and dimensionless quantity proportional to the erythemally weighted UV irradiance. It provides an estimate of the potential for skin damage. Equation (5) describes its calculation method:

(5) $$I_{UV}=\;k_{er}\int_{250\;nm}^{400\;nm}E_{\lambda }S_{er}(\lambda )d\lambda$$

where *I_UV_* is the UV index, $k_{er}$ is a scaling factor equal to 40 $\frac{m^{2}}{W}$, $E_{\lambda }$ is the solar spectral irradiance expressed in $\frac{W}{m^{2}nm}$, $S_{er}$ is the erythema action spectrum and *λ* is the wavelength in nm. To obtain a quantitative parameter for UV measurements, the concept of effective irradiation ($E_{eff}$) is introduced, describing the amount of energy per m^2^ in the electromagnetic spectrum ranging from 400 to 250 nm, calculated using Equation (6). For practical purposes, both *I_UV_* and $E_{eff}$ are included in the results.

(6) $$E_{eff}=\frac{I_{UV}}{0.025\;\frac{m^{2}}{W}}$$

#### 2.5.5. Energy Balance Calculation

Sunlight radiation was measured using the Global Solar Atlar website in the specific coordinates of the experiment, using the DNI value to estimate the energy per m^2^ that was available to capture in Thunder Bay; this value is 4.141 $\frac{kWh}{m^{2}}$. The size of each mirror was measured as 0.425 m^2^, forming a total area of 0.85 m^2^. By correlating the collection area and the reaction time, we obtain a total of 483.9 kW of energy flow from the sun going into the reactor. Nevertheless, this estimation is approximate, since some energy is unavoidably lost through cloud interference and imperfect mirror reflectance. To calculate the amount of energy that was reflected, we measured and recorded the inner surface temperature of the reactor using a thermocouple during each experiment, achieving a maximum temperature of 145.1 °C after 55 min of reaction. Considering that the material of the surface of the reactor is stainless steel, the ΔH was obtained using Equation (7):

(7) ΔH= mCpΔT

where ΔH is the amount of energy in the form of enthalpy that is getting into the system, m is the amount of material that is being heated, in this case, stainless steel, Cp is the heating capacity of the material, and ΔT is the change in temperature that the system presents during the heating process. Based on our calculations, only 52.72 kW were used by the solar reactor for the reaction due to the correlation between the reactor’s surface temperature increase and the energy exchange shown in Equation (7). With this information, the actual temperature of the fluid can be calculated using the resistance analysis for heat transfer considering a two-layer conduction system, expressed in Equation (8):

(8) $$T_{\infty }=\;T_{s}-q\left(\frac{\Delta X_{A}}{k_{A}A_{A}}+\frac{\Delta X_{B}}{k_{B}A_{B}}\right)$$

where $T_{\infty }$ is the internal temperature of the glass layer in the conduction system, between the liquid surface of the sample and the solid surface of the glass tube; $T_{s}$ is the external temperature of the silicone layer of the conduction system, which is being measured with the thermocouple; *q* is the energy flow, which is dependent on the experiment’s sunlight; ΔX*_i_* is the thickness of the layer; *k_i_* is the thermal conductivity constant that depends on the capability of a material to transfer heat; and *A_i_* is the cross-sectional area, which depends on the geometry of the layer. Equation (8) shows the temperature difference inside each layer of the reactor, where *T_s_* is the maximum temperature in the outer layer, but due to the energy conduction capacity of each material, the temperature is supposed to be different and should be considered to obtain an accurate result.

#### 2.5.6. Catalyst Reusability Test

A 30 mL RhB solution with an initial concentration of 100 ppm and a pH of 2.7 was prepared with 10 mg of APS and MW biochar loaded with MnO_2_, and 150 uL of H_2_O_2_. The first run lasted 30 min, taking samples every 5 min. After the run, all material residues were collected, including tips, beakers, glass tube and cuvettes, as the materials contained small catalyst particles both diluted in water and stuck on its surface. Therefore, every liquid was poured into a 250 mL glass beaker and each material was washed with distilled water, which was also poured into the same 250 mL glass beaker. Finally, the solution was put into an oven at 105 °C for 12 h. After recovering the solid-phase biochar, it was smashed and weighed to calculate the recovery yield. Then, the recovery yield was used as a reference to adjust the actual loading of RhB in the next run to contain an equivalent mass ratio of RhB versus biochar. The process of recovery was repeated after every run, for a total of three runs.

## 3. Results and Discussion

### 3.1. Comparison Between Solar and UV Fenton Systems

**Figure 1:**
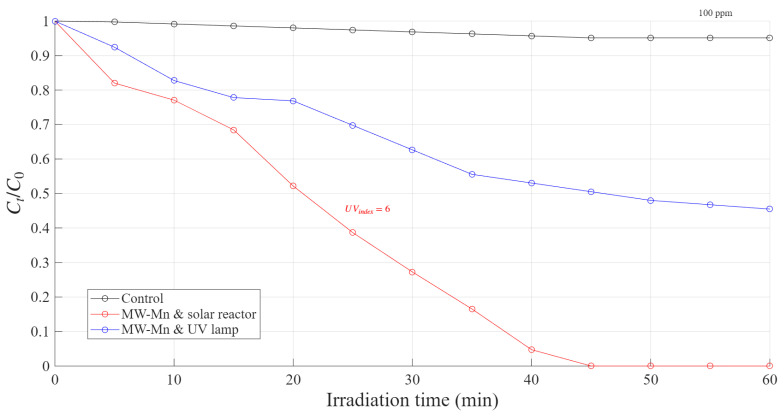
Comparison of the degradation behavior of RhB using different reactors, catalyzed by a MW-Mn catalyst under solar irradiation ($E_{eff}$ = 240 $\frac{W}{m^{2}}$) and UV lamp conditions.

Comparing solar collector and UV Fenton systems is crucial to determine how effectively natural sunlight can substitute artificial UV radiation for driving advanced oxidation processes, thereby assessing both the environmental and energy-efficiency advantages of solar-based treatment systems. As shown in Figure 1, the degradation of RhB in the solar reactor system under the same conditions was much faster than when using the UV reactor. This result represents an improvement in the reaction’s efficiency associated with the interaction of the system and all sunlight wavelengths. These findings indicate the potential for redesigning advanced oxidation processes in research and industrial applications, transitioning from artificial UV systems to efficient sunlight-based capture technologies. This represents only a preliminary indication of feasibility.

### 3.2. Catalyst Characterizations

#### 3.2.1. X-Ray Diffraction (XRD) Analysis

**Figure 2:**
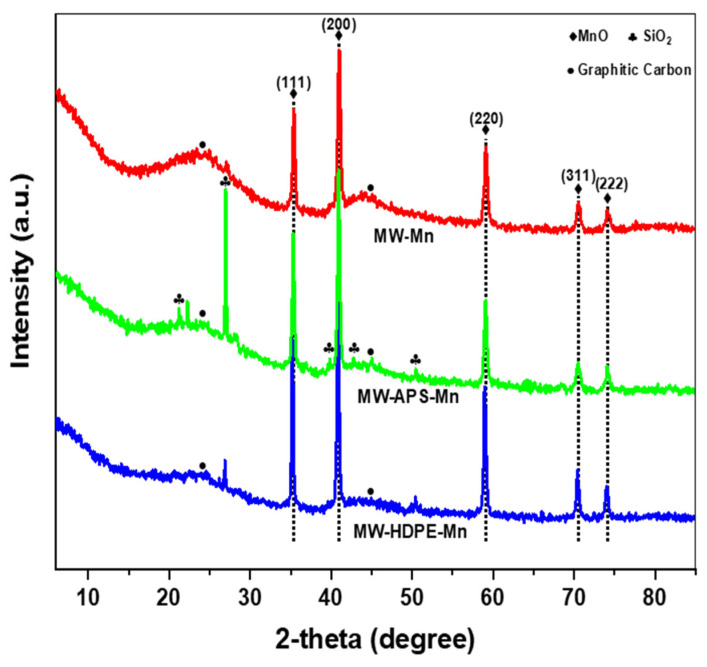
XRD analysis of three different Mn-loaded biochar catalysts.

The XRD analysis of MW-Mn, MW-HDPE-Mn, and MW-APS-Mn are interpreted in Figure 2. All three samples demonstrate sharp diffraction peaks at 2θ positions of 35.4°, 40.9°, 59.1°, 70.5°, and 74.2°, representing the (111), (200), (220), (311) and (222) crystal planes of MnO, respectively (JCPDS 75–0626) [18,19]. Therefore, it is evident that Mn is loaded successfully at the catalyst surfaces. Around 2θ ≈ 24°, a broad diffraction peak is visible corresponding to the (002) plane, which is attributed to amorphous graphitic carbon [20]. The MW-Mn sample also showed a more prominent diffraction peak at 2θ ≈ 44°, indicating the transformation into graphitic carbon [21]. In the MW-APS-Mn catalyst, additional diffraction peaks at 2θ of 21.2°, 26.9°, 39.8°, 42.7°, 50.4° are attributed to the presence of quartz as SiO_2_ crystal in the catalyst as a major component of paper sludge [22]. Due to high temperature (800 °C), the characteristic peaks for other paper sludge components like CaCO_3_ are weakly reflected in the MW-APS-Mn catalyst [23]. The high pyrolysis temperature and substantial proportion of MW in the MW-HDPE-Mn make the expected peaks for semi-crystalline HDPE at 2θ ≈ 21° and 24° disappear with prominent diffraction peaks for successful Mn-doping. Similar observations have also been found in previous studies [24,25].

#### 3.2.2. Fourier Transform Infrared (FTIR) Analysis

**Figure 3:**
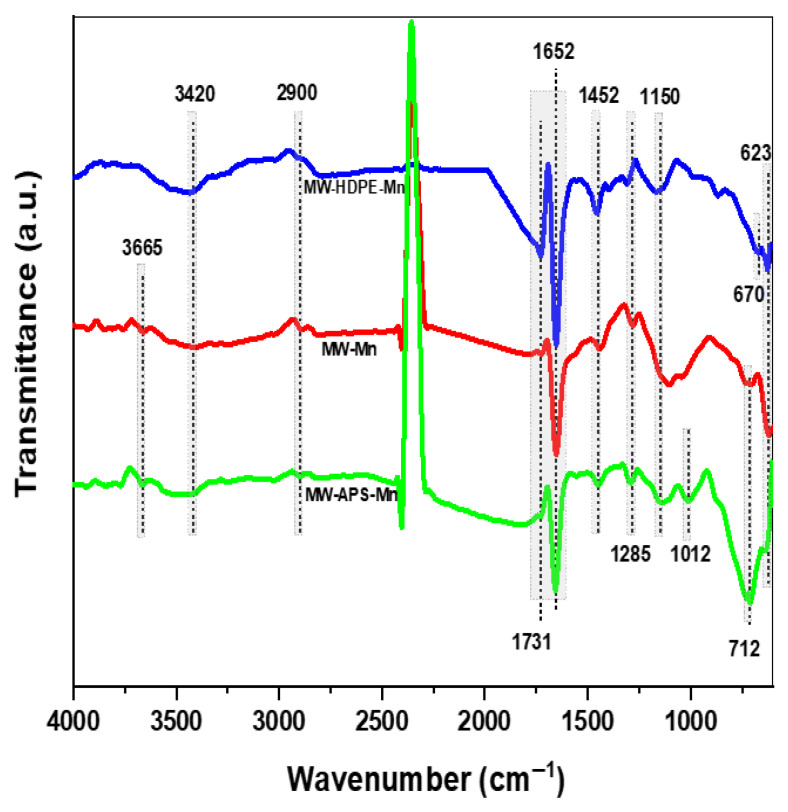
FTIR analysis of three different Mn-loaded biochar catalysts.

FT-IR analysis of three catalysts (MW-Mn, MW-HDPE-Mn, and MW-APS-Mn) was performed to observe the surface functional groups of the Mn-loaded biochar catalysts (Figure 3). In all three-catalyst samples, the peaks around 3665 cm^−1^ and 3420 cm^−1^ were attributed to -OH stretching vibrations [26,27]. The peaks around 2900 cm^−1^ indicate C-H stretching [24]. Prominent peaks within the region between 1600 cm^−1^ and 1750 cm^−1^ in all three catalysts are ascribed to aliphatic or aromatic groups like C=C, and carbonyl C=O stretching vibration [27,28]. Phenolic (C–OH) groups are depicted around 1452 cm^−1^ [29]. Peaks within the regions of 1250–1335 cm^−1^ represent C-N stretching, and phenolic C–O bonds are clearly observed around 1150 cm^−1^ [26,30]. An unique peak around 1012 cm^−1^ was found in the case of the MW-APS-Mn catalyst only, which might be attributed to C–O bonds or Si–O–Mn that aligns well with the previous study [27]. This finding correlates with the presence of Si in the XRD and EDX analysis of the MW-APS-Mn catalyst sample. The successful Mn loading of all three catalysts was assured with the presence of Mn-O stretching and bending around 623 cm^−1^, 670 cm^−1^, and 712 cm^−1^, respectively [31,32]. All these findings on the FTIR spectra are in good alignment with the XRD and EDX analysis of all three Mn-loaded biochar catalysts.

#### 3.2.3. Surface Morphology and Elemental Distribution Analysis

**Figure 4:**
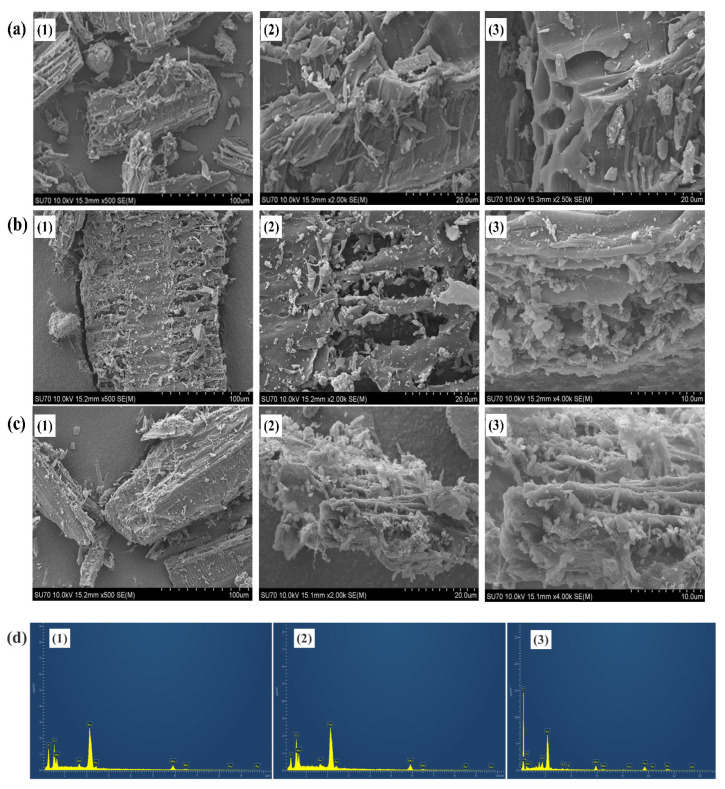
SEM images of Mn-loaded biochar catalyst: (**a**) MW-Mn, (**b**) MW-HDPE-Mn, (**c**) MW-APS-Mn and (**d**) (1)–(3) corresponding EDS spectra.

To observe the morphological structure and localized elemental distribution, all catalysts were characterized using SEM-EDS. As shown in Figure 4a, the MW-Mn catalyst showed pore-containing prominent vessel structures in several layers and relatively smoother surface when compared with MW-HDPE-Mn. The surface of MW-Mn was also seen to be irregularly loaded with small agglomerated granular particles, which might be due to the presence of manganese oxide particles. A similar trend was seen in another Mn-loaded biochar-related study [33]. In Figure 4d(1), EDS analysis of the MW-Mn catalyst showed the atomic percentage of C (52.99%) as a dominant element confirming the transformation to a carbon-rich material through high pyrolysis temperature. In addition, Mn (22.84%) and O (24.18%) are present in significant quantities, which perfectly aligns with the SEM results and justifies the XRD crystalline peaks for Mn-loaded biochar catalysts. Figure 4b shows the SEM images of the fractured surfaces of the MW-HDPE-Mn biochar catalyst. The regular pore-containing surface structure shown in the MW-Mn biochar catalyst disappeared when co-pyrolysis of MW and HDPE was performed to prepare the MW-HDPE-Mn biochar catalyst. The molten HDPE may be occluded within the pores of MW particles in the MW-HDPE-Mn catalyst and distributed through the surface evenly. HDPE not only occupies the pores of MW but also acts as a binder to hold the MW particles firmly. Therefore, the surface of the MW-HDPE-Mn biochar catalyst did not show significant pores, which is like the SEM image of HDPE/Poplar biochar presented by Zhang et al. [24]. Like the MW-Mn biochar catalyst, small granule-like agglomerated particles appeared on the biochar surface appeared, which indicates that the Mn was loaded successfully. The EDS analyses also confirmed Mn-loading in the MW-HDPE-Mn biochar catalyst with a high Mn elemental atomic percentage (33.51%) and percentage of O (29.20%). It was also shown to be a carbon-rich material; a dominant presence of C (37.28%) is evident in Figure 4d(2). Magnifications of MW-APS-Mn in Figure 4c showed a rough, irregular surface with cracks and scattered fine particles, revealing the presence of Si and Ca on catalyst surface as these elements are dominant in paper sludge. As with the other two catalysts, the surface of the MW-APS-Mn catalyst was also observed to be covered with aggregated manganese oxide particles, proving a successful Mn-loading. However, all these mineral particles are sufficient to infiltrate the surface pores, and thus are not predominantly seen. Similar observations were found in the existing literature [34]. EDS analysis of the MW-APS-Mn biochar catalyst in Figure 4d(3) showed the atomic percentage of C (83.85%), O (13.91%), Mn (1.31%) and small amount of Si and Ca, incorporated with the results of SEM and XRD analysis.

### 3.3. Effect of the Initial Dye Concentration and Catalyst Type

**Figure 5:**
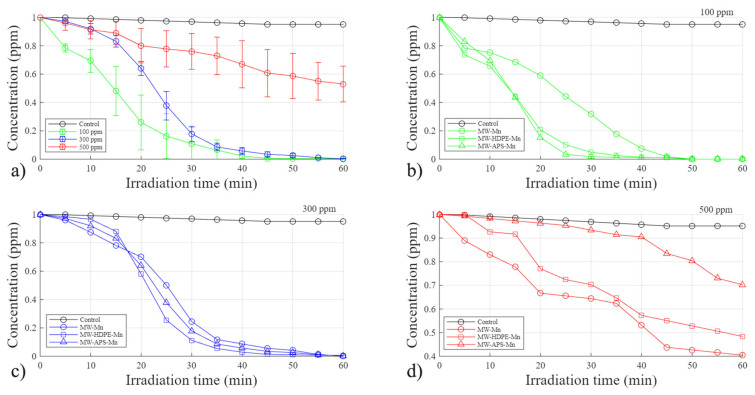
Effects of different dye concentrations on removal efficiency: (**a**) Overall average of the experiments (**b**) 100 ppm; (**c**) 300 ppm; and (**d**) 500 ppm.

To study the effect of the initial dye concentrations, nine different experiments were conducted using three catalysts and three different initial concentrations, and the results are provided in Figure 5. As shown in Figure 5a, based on the average results, regardless of the initial concentrations, a significant standard deviation was observed due to the change in sunlight conditions (UV indexes) and type of catalysts used. However, the results still represent a general trend of progress in the reaction. A general conclusion here is that within the 60 min reaction time, our system cannot completely degrade the dye at 500 ppm, regardless of the catalyst used. However, it was able to achieve 100% degradation of dye at 300 ppm. Therefore, 500 ppm concentration was found to be the upper degradation capacity limit within our system.

Figure 5b presents a comparison between the catalysts’ effects under the 100 ppm concentration of RhB. Here, the most efficient catalyst was MW-APS-Mn, which showed a complete degradation in only 30 min of reaction, followed by the MW-HDPE-Mn catalyst. Figure 5c shows the results when the initial RhB concentration was increased to 300 ppm. All the catalysts demonstrate similar behavior, but the most efficient was MW-HDPE-Mn with a complete RhB degradation within 50 min, followed by MW-APS-Mn. However, under a higher initial concentration of RhB of 500 ppm in Figure 5d, within the 60 min of reaction where data was collected, no complete degradation was obtained with any of the catalysts, indicating that with the tested catalyst loading, 500 ppm of RhB exceeded the limit of the degradation system. Here, the most efficient catalyst was found to be MW-Mn, followed by MW-HDPE-Mn and finally MW-APS-Mn.

Interestingly, for the MW-APS-Mn catalyst, at low initial RhB concentration (100 ppm), it exhibited the highest degradation efficiency. However, as the dye concentration increased, its performance reduced significantly. This limitation is most likely attributed to an insufficient number of accessible active sites to support efficient degradation at higher substrate loadings. This behavior suggests that MW-APS-Mn may possess a smaller number of active surface sites.

On the other hand, the MW-Mn catalyst likely contains the highest density of active sites. However, under low RhB concentrations (100 ppm, as shown in Figure 5b), the degradation appeared to be limited by mass transfer. In such conditions, RhB molecules may adsorb onto active sites that are not in proximity to hydroxyl radicals, reducing the likelihood of effective radical–dye interactions and slowing the degradation process. At higher RhB concentrations, more dye molecules are available to occupy active sites, increasing the probability of contact with hydroxyl radicals and thereby enhancing degradation efficiency.

### 3.4. Reaction Kinetics of Different Catalysts

#### 3.4.1. Reaction Kinetics Studied Using MW-Mn Catalysts

Determining the reaction kinetics is essential to verify that the reaction proceeds as intended. However, modifications to the system may alter its expected behavior, as frequently reported in studies conducted under UV-based systems. Therefore, a comprehensive kinetic analysis was performed using different catalysts within the solar Fenton system to provide insights into the reaction mechanisms and potential limitations.

**Figure 6:**
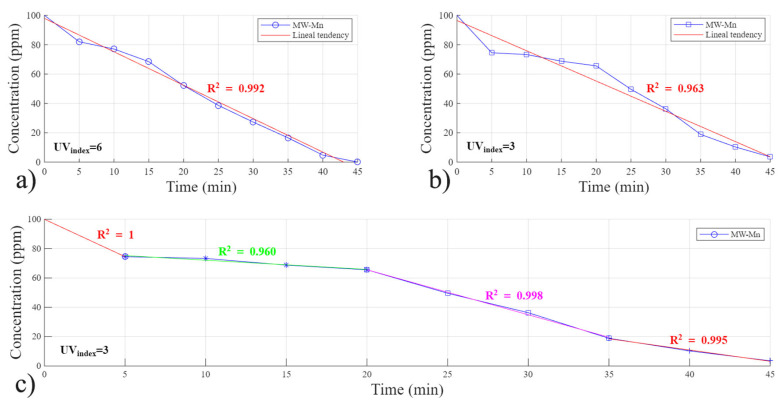
Reaction kinetics study for MW-Mn catalyst in solar Fenton reactor: (**a**) Zero-order fit at a UV index of 6 ($E_{eff}$ = 240 $\frac{W}{m^{2}}$), (**b**) Zero order fit at UV index of 3 ($E_{eff}$ = 120 $\frac{W}{m^{2}}$), and (**c**) four-step all zero-order linear model at a UV index of 3 ($E_{eff}$ = 120 $\frac{W}{m^{2}}$).

Figure 6 shows the experimental results using a MW-Mn catalyst and 100 ppm of RhB; both experiments were conducted under the same concentration of reactants and catalyst. The kinetics analysis presents an almost perfectly linear behavior (Equation (2)). A zero-order fit suggests that the reaction rate is limited by factors other than dye concentration, such as: (1) saturation of catalytic sites on the biochar surface; (2) constant generation of hydroxyl radicals (•OH) that do not depend on dye concentration; and (3) mass transfer or light intensity control, rather than chemical kinetics, dominating the rate. The experiments show different R^2^ values when impacted by lighting conditions, where their effects include the rate of the reaction. Specifically, as seen when comparing Figure 6a,b, the overall order of the reaction is impacted by the UV index. When the UV index was reduced from 6 to 3 in Figure 6b, the fitting of the reactions using zero-order kinetics became less accurate (R^2^ decreased from 0.996 to 0.963).

In Figure 6b, we identify four main linear stages, and they are further fitted using a zero-order model for each step and displayed in detail in Figure 6c. During the first 5 min, the high number of active sites available in the biochar and the temperature increase of the reactor lead to a rapid mass transfer of free hydroxyl radicals and the RhB molecules, resulting in fast degradation. Then, the following 15 min (5 to 20 min) represent a slow stage, where gas bubbles are starting to emerge, coming out from the vessel’s surface due to a gas produced in the reaction. This probably creates a phase barrier and slows down the reaction between RhB and the hydroxyl radicals. In the third stage (20–35 min), when the maximum temperature is reached and water is boiling, the system is considered to reach a not-fast reaction state, leading to a constant degradation of the dye for the following 15 min. In this stage, the negative impact of the phase barrier is suppressed by the positive impacts of the increased reaction temperature. During the final stage (35–45 min), the reaction rate decreases relative to the previous stage. This slowdown is attributed to the depletion of dye molecules and the consequent reduction in occupied active catalytic sites. Although the reaction exhibits zero-order behavior under sufficient reactant concentration, the rate naturally declines once these conditions are no longer maintained, consistent with the typical transition observed as zero-order systems approach completion.

#### 3.4.2. Reaction Kinetics Studied Using MW-HDPE-Mn Catalysts

**Figure 7:**
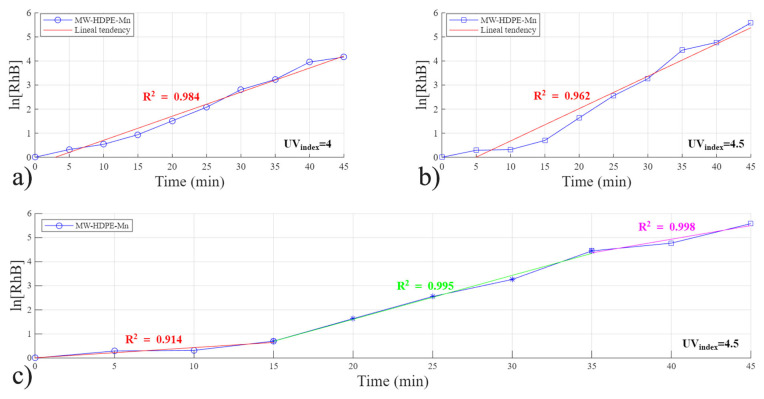
Reaction kinetics study for the MW-HDPE-Mn catalyst: (**a**) pseudo-first-order fit at a UV index of 4 ($E_{eff}$ = 160 $\frac{W}{m^{2}}$), (**b**) pseudo-first-order fit at a UV index of 4.5 ($E_{eff}$ = 180 $\frac{W}{m^{2}}$), and (**c**) a three-step all pseudo-first-order linear model at a UV index of 4.5 ($E_{eff}$ = 180 $\frac{W}{m^{2}}$).

For the MW-HDPE-Mn catalyst, the results of the kinetics study are presented in Figure 7, which shows a comparison between two experiments using an initial RhB concentration of 100 ppm and the same concentration of reactants and catalyst. For this catalyst, the linear relationship was best represented as a pseudo-first-order reaction; the reaction could not be accurately described as either zero-order or second-order. When the Fenton reaction follows a pseudo-first-order kinetic model, it indicates that the degradation rate of RhB depends directly on its concentration, while the concentrations of hydroxyl radicals and hydrogen peroxide remain effectively constant throughout the reaction. This behavior suggests that the reaction is mainly controlled by the chemical oxidation process rather than other process factors such as light intensity or mass transfer. Under these conditions, the degradation follows an exponential decay trend, meaning that the rate decreases as the dye concentration reduces. The pseudo–first order fit also implies that the system maintains a steady generation of reactive radicals, which continuously attack the dye molecules, making this kinetic behavior typical for well-optimized photo-Fenton and solar Fenton processes.

The pseudo-first order is explained previously in Equation (3); however, the natural logarithm used in the equation affects the concentration analysis, where the smaller the Y-axis value in the plot, the more concentrated the solution. Figure 7a shows a lineal one-stage process and Figure 7b presents a one-stage process with a lower R^2^ value. Considering an almost equal UV index of these two runs (4 in run 1 vs. 4.5 in run 2), the difference in light strength is associated with the presence of clouds during the experiment, which affects the maximum temperature, which in the Figure 7a experiment was 123.9 °C, while Figure 7b experiment’s maximum temperature was 99.4 °C. This indicates that the light source is acting as a limiting step and allows us to analyze the reaction like a three-lineal-stage process, as plotted in Figure 7c. The first 15 min of the reaction represents the heating stage; it presents an almost constant slope due to the slowly increasing reactor temperature, reaching only 86.3 °C. The second stage lasts 20 min (15 to 35 min); the temperature increases to 97 °C and remains almost constant, leading to a rapid degradation and representing the most important stage of dye degradation; the mass transfer is behaving correctly and no limiting steps can be identified. Then, the final 10 min (35–45 min) presents a decrease in the rate of reaction due to the reduced availability of RhB molecules to be reacted. Specifically, by the time of 35 min, only 1.16 ppm remain in the system, decreasing to 0.37 ppm by 45 min.

#### 3.4.3. Reaction Kinetics Studied Using MW-APS-Mn Catalysts

**Figure 8:**
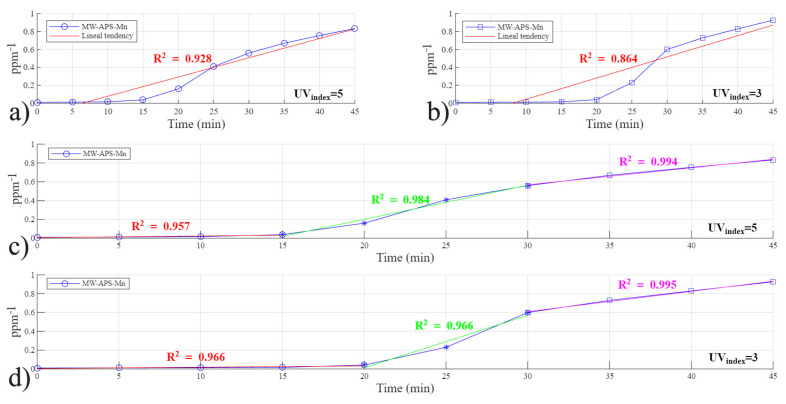
Reaction kinetics study for the MW-APS-Mn catalyst: (**a**) second order fit at UV index of 5 ($E_{eff}$ = 200 $\frac{W}{m^{2}}$), (**b**) second order fit at a UV index of 3 ($E_{eff}$ = 120 $\frac{W}{m^{2}}$), (**c**) three-step all second-order linear model at a UV index of 5 ($E_{eff}$ = 200 $\frac{W}{m^{2}}$), and (**d**) three-step all second-order linear model at UV index of 3 ($E_{eff}$ = 120 $\frac{W}{m^{2}}$).

The results for the last type of catalyst used in this study, MW-APS-Mn, are presented in Figure 8. The behavior of the dye degradation in this system does not present a highly linear behavior in any of the analysis equations; however, the second-order kinetics described in Equation (4) presents a higher R^2^ value considering a one-lineal-stage system, as shown in Figure 8a,b. These findings indicate that the MW-APS-Mn system operates under conditions where both dye concentration and active site availability play significant roles, supporting a chemisorption rate-limiting mechanism. This kinetic behavior aligns with adsorption-driven photo-Fenton degradation typically observed in catalysts with limited accessible reactive sites, leading to a limited amount of RhB molecules that can attach to the catalyst surface. In this case, using a 100 ppm concentration, a significantly rapid degradation is observed even though it shows second-order behavior; however, the reaction should become slower as the initial concentration is increased; this hypothesis is demonstrated experimentally. Again, to improve the R^2^ value, a three-stage process was considered and fitted to the data in Figure 8c. In this case, data from two runs conducted under different sunlight conditions are expressed in Figure 8c and Figure 8d, respectively. Comparing Figure 8c,d, a noticeable change in the duration of the first stage can be observed; in Figure 8c, this lasted from 0 to 15 min, while in Figure 8d, the first stage is 5 min longer (from 0 to 20 min) and maintains the same degradation rate, this was primarily because the UV index did not change significantly, and it represents the heating-up stage of the reactor. The second stage in both Figure 8c,d holds the fastest degradation reaction speed. It takes the highest slope but lasts only between 10 and 15 min. The final stage, in both Figure 8c,d, is associated with the low RhB concentration that is still in the liquid phase and has not been absorbed yet, leading to a decreased degradation rate reflected by the reduced slope.

### 3.5. Prediction of Reactor Temperature and Dye Degradation Efficiency

**Figure 9:**
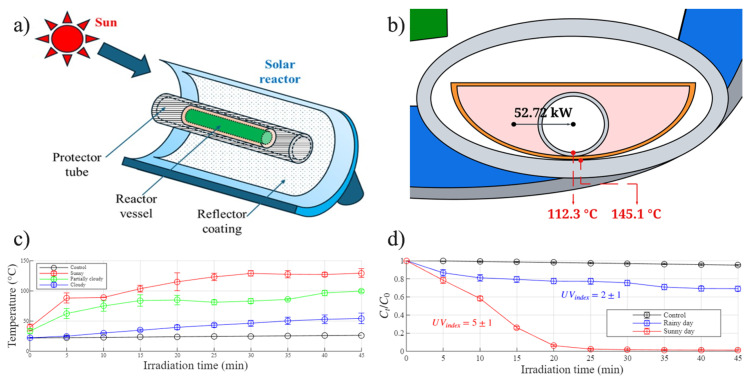
(**a**) Simplified structure schematics of the solar reactor, (**b**) layers representation and temperature difference within the reactor tube, (**c**) maximum surface temperature of the solar reactor under varying weather conditions, and (**d**) the correlation between UV intensity and RhB degradation efficiency using the MW-Mn catalyst ($E_{eff}$_rainy_ = 200 ± 40 $\frac{W}{m^{2}}$, $E_{eff}$_sunny_ = 80 ± 40 $\frac{W}{m^{2}}$).

The results of the experimental data used in the energy balance calculations are shown in Figure 9. In Figure 9a, an illustration of the reactor structure in the system is presented, where the heating is due to the radiation from the sun; however, the cloud represents the weather conditions that cannot be controlled and will influence the energy flow. Other uncontrollable factors are ambient temperature and wind. Figure 9b is a representative energy flow model of the solar reactor under the highest temperature experiment reported for the date of 14 August 2025, where the energy is affecting the reaction directly after the effects of the reactor’s efficiency. The figure also shows the temperature change because of the different layers and where exactly these temperatures are located. Internally, the reactor presents two layers that separate the temperature measuring spot and the reaction solution. The two red dots shown in Figure 9b represent where both temperatures are measured. In the outer layer (below the orange-colored silicone boat), indicated by a temperature of 145.1 °C, the thermocouple is placed, and the temperature is measured, followed by the orange silicone boat layer, and a transparent glass tube layer. Between this last layer and the liquid surface of the reaction, the second inner layer temperature is measured as 112.3 °C. In Figure 9c, the reaction temperature profiles are grouped into three distinct trends, which are mainly influenced by variations in weather conditions. These profiles reflect the approximate surface temperature of the solar reactor under typical environmental conditions in Thunder Bay, ON, Canada. In Figure 9d, the effects of lighting intensity and reactor surface temperature are correlated with the dye degradation efficiency using a 100 ppm initial RhB concentration using the MW-Mn catalyst. The results suggest that the optimal degradation performance is achieved on sunny days, particularly when the UV index is ≥6 and the reactor surface temperature ranges between approximately 120 °C and 140 °C. This correlation provides valuable insight into both the energetic behavior of the system and the practical applicability of solar energy for solar-assisted water treatment. It should be noted that the *Beer–Lambert* approach assumes negligible interference from intermediate species, which may introduce some uncertainty in the concentration estimation during the degradation process.

### 3.6. Effects of Sunlight Spectrum Filtration

**Figure 10:**
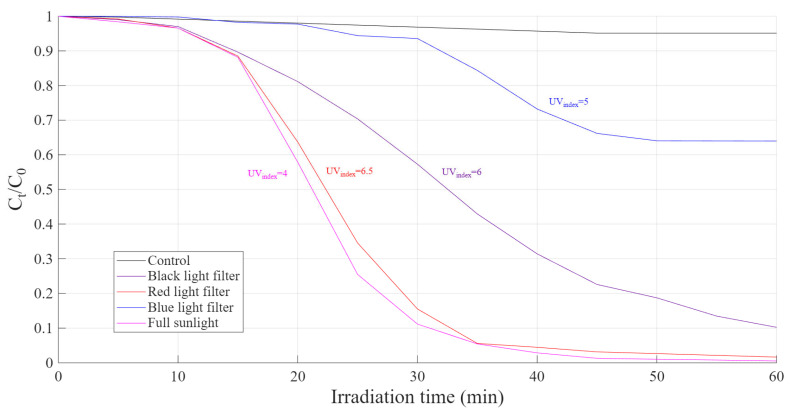
Effects of light filters on RhB degradation ($E_{eff}$_black_ = 160 $\frac{W}{m^{2}}$, $E_{eff}$_red_ = 260 $\frac{W}{m^{2}}$, $E_{eff}$_blue_ = 240 $\frac{W}{m^{2}}$, $E_{eff}$_full_ = 200 $\frac{W}{m^{2}}$).

The effects of light filters on RhB degradation are shown in Figure 10. Full-spectrum sunlight exhibited the highest RhB degradation efficiency, confirming the strong contribution of the broad wavelength range present in natural solar radiation. When a red-light filter was applied, only a slight decrease in performance was observed, suggesting that longer wavelengths (≥600 nm) still contribute to photo activation of the catalyst, although less effectively than the full spectrum. In contrast, the use of a black-light filter led to a marked reduction in degradation efficiency, indicating that restricting the spectrum primarily to near-UV and violet light limit the generation of reactive oxygen species (ROS) to some extent. The most pronounced negative effect was observed with the blue-light filter. However, it should be emphasized that the blue-filter experiment was conducted under a lower UV index (approximately 4), compared to the UV index range of 5–6.5 during the other experiments. Therefore, part of the performance reduction may be attributed to reduced UV intensity rather than the exclusion of longer wavelengths alone.

Overall, these observations indicate that while UV radiation plays a critical role in driving photo-Fenton reactions, the synergistic contribution of visible light, particularly in the green-to-red regions, enhances ROS generation and accelerates RhB degradation under full-spectrum sunlight.

### 3.7. Reusability in MW-APS Biochar Catalyst

**Figure 11:**
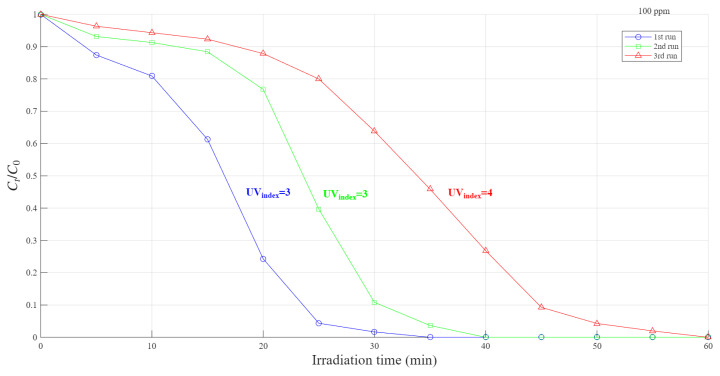
Dye degradation under a three-run reused MW-APS-Mn catalyst ($E_{eff}$_first_ = 120 $\frac{W}{m^{2}}$, $E_{eff}$_second_ = 120 $\frac{W}{m^{2}}$, $E_{eff}$_third_ = 160 $\frac{W}{m^{2}}$).

Although the catalysts developed here can be highly efficient, to optimize their utilization process, it is essential to design a recovery process that minimizes particle loss and to evaluate whether the catalyst can be reused multiple times without significant loss of performance. Figure 11 shows the degradation results of a three-run reused MW-APS-Mn catalyst. Due to mass losses that occurred during separation, after the first run, only 86% of the catalyst mass recovered, decreasing to 51% after the second run. Based on the starting catalysts masses, initial concentrations of the RhB were adjusted using the recovery yield as a reference. Across the reuse experiments, complete RhB removal was achieved within the first three catalytic cycles.

However, a noticeable decline in reaction rate was observed, with the second cycle proceeding 1.14 times slower and the third cycle 1.71 times slower than the first. This decrease in catalytic efficiency may be attributed to the partial deactivation of active sites during the reaction. Such deactivation could result from (i) chemical interaction between RhB molecules and reactive surface functionalities, or (ii) the chemisorption of dye molecules and/or their degradation intermediates onto active sites that were not fully removed by the applied regeneration procedure (water washing followed by drying). Overall, these results suggest that while the catalyst can be reused effectively at least twice, its performance longevity could be improved by developing a more energy-efficient reactivation method to restore active sites.

## 4. Conclusions

In this study, MnO_2_-modified biochar catalysts derived from maple wood and waste-based feedstocks (APS and HDPE) were demonstrated to be effective heterogeneous photo-Fenton catalysts for solar-driven degradation of Rhodamine B under real, full-spectrum sunlight using a simple solar reactor. Compared with a 365 nm UV system, the solar reactor achieved faster degradation, highlighting the advantage of utilizing the full solar spectrum. Complete RhB removal was achieved at concentrations of 100–300 ppm, while 500 ppm exceeded the catalytic capacity within the tested time, revealing concentration-dependent limitations related to active-site availability and mass transfer. Distinct kinetic behaviors were observed among the catalysts, emphasizing the strong influence of biochar composition and MnO_2_ dispersion on reaction pathways and underscoring the inadequacy of assuming a universal kinetic model for solar photo-Fenton systems. Degradation efficiency correlated strongly with solar irradiance and reactor temperature, with optimal performance under high UV index conditions, confirming synergistic contributions from both UV and visible light. Although the catalysts retained activity over multiple cycles, progressive rate decay and recovery losses highlight the need for improved catalyst stability and separation strategies to enable scalable, low-energy solar wastewater treatment applications.

## Data Availability

The raw data required to reproduce these findings are available upon request.

## Abbreviations

The following abbreviations are used in this manuscript:

RhB	Rhodamine B
UV	Ultraviolet
AOPs	Advanced Oxidation Processes
ROS	Reactive oxygen species
MW	Maple wood
APS	Aerobic paper sludge
HDPE	high-density polyethylene
XRD	X-ray diffraction
FTIR	Fourier transform infrared
SEM	Scanning electron microscopy
EDS	Energy-dispersive spectroscopy
IMDA	Integral Method of Data Analysis

## References

[1] Al-Tohamy R., Ali S.S., Li F., Okasha K.M., Mahmoud Y.A.G., Elsamahy T., Jiao H., Fu Y., Sun J. (2022). A critical review on the treatment of dye-containing wastewater: Ecotoxicological and health concerns of textile dyes and possible remediation approaches for environmental safety. Ecotoxicol. Environ. Saf..

[2] Amalina F., Abd Razak A.S., Krishnan S., Zularisam A.W., Nasrullah M. (2022). A review of eco-sustainable techniques for the removal of Rhodamine B dye utilizing biomass residue adsorbents. Phys. Chem. Earth.

[3] Yalasangi V., Mayilswamy N., Kandasubramanian B. (2024). Biochar-derived adsorbents for removal of Rhodamine B from wastewater. Bioresour. Technol. Rep..

[4] Bushra R., Mohamad S., Alias Y., Jin Y., Ahmad M. (2021). Current approaches and methodologies to explore the perceptive adsorption mechanism of dyes on low-cost agricultural waste: A review. Microporous Mesoporous Mater..

[5] Zhang X., Zhang M., Liu H., Gu J., Liu Y. (2019). Environmental sustainability: A pressing challenge to biological sewage treatment processes. Curr. Opin. Environ. Sci. Health.

[6] Kumari P., Kumar A. (2023). Advanced oxidation process: A remediation technique for organic and non-biodegradable pollutant. Results Surf. Interfaces.

[7] dos Santos C.R., Rodrigues A.C., Vilasbôas F.d.S., Mistura C.M., Schneider I.A.H. (2025). On the Fenton’s process for the treatment of effluents from the dyeing of agates containing Rhodamine B and ethanol. Discov. Chem. Eng..

[8] Zhang Y., Shaad K., Vollmer D., Ma C. (2021). Treatment of textile wastewater using advanced oxidation processes—A critical review. Water.

[9] Xu S.-L., Wang W., Song Y., Tang R., Hu Z.-H., Zhou X., Yu H.-Q. (2025). Expanding the pH range of Fenton-like reactions for pollutant degradation: The impact of acidic microenvironments. Water Res..

[10] Cuong D.V., Wu P.-C., Chen L.-I., Hou C.-H. (2021). Active MnO_2_/biochar composite for efficient As(III) removal: Insight into the mechanisms of redox transformation and adsorption. Water Res..

[11] Angaru G.K.R., Pal C.A., Lingamdinne L.P., Husain Z.M., Kulkarni R., Choi Y.-L., Koduru J.R., Chang Y.-Y. (2024). High-performance MnO_2_ embedded fly ash zeolite applied for effective mineralization of bisphenol-A and sorption of Congo red: Mechanism, real water application, and toxicity assessment. Chem. Eng. Sci..

[12] Laishram D., Kim S.B., Lee S.Y., Park S.J. (2025). Advancements in biochar as a sustainable adsorbent for water pollution mitigation. Adv. Sci..

[13] Zhang Y., Sun X., Bian W., Peng J., Wan H., Zhao J. (2020). The key role of persistent free radicals on the surface of hydrochar and pyrocarbon in the removal of heavy metal–organic combined pollutants. Bioresour. Technol..

[14] Martin-Montero A., Zapanti A.M., Pliego G., Casas J.A., Garcia-Costa A.L. (2025). Solar photo-Fenton: An effective method for MCPA degradation. Processes.

[15] Brillas E. (2023). Solar photoelectro-Fenton: A very effective and cost-efficient electrochemical advanced oxidation process for the removal of organic pollutants from synthetic and real wastewaters. Chemosphere.

[16] Tran H.D., Nguyen D.Q., Do P.T., Tran U.N. (2023). Kinetics of photocatalytic degradation of organic compounds: A mini-review and new approach. RSC Adv..

[17] Amakiri K.T., Angelis-Dimakis A., Ramirez Canon A. (2022). Recent advances, influencing factors, and future research prospects using photocatalytic process for produced water treatment. Water Sci. Technol..

[18] Fu Q., Xu X., Miao R., Wang M., Zhou H., He L., Guan Q. (2022). Mn-embedded porous rubber seed shell biochar for enhanced removal of copper ions and catalytic efficacy of the used adsorbent for hydrogenation of furfural. Chem. Eng. J..

[19] Du J., Gao L., Yang Y., Chen G., Guo S., Omran M., Chen J., Ruan R. (2021). Study on thermochemical characteristics properties and pyrolysis kinetics of the mixtures of waste corn stalk and pyrolusite. Bioresour. Technol..

[20] Zhang J., Li Q., Zhang J., Liu H., Wang H., Zhang J. (2025). Enhanced CO_2_ absorption in amine-based carbon capture aided by coconut shell-derived nitrogen-doped biochar. Sep. Purif. Technol..

[21] Tomin O., Yazdani M.R. (2022). Production and characterization of porous magnetic biochar: Before and after phosphate adsorption insights. J. Porous Mater..

[22] Zhang X., Tong G., Zhou Y., Li G., Zhang H. (2021). Enhancing paper sludge dewatering by waste polyester fiber and FeCl_3_ for preparation of Fe-rich biochar. BioResources.

[23] Niu J., Fan S., Wu Z. (2025). Preparation of biochar from papermaking sludge and its adsorption characteristics for tetracycline. Toxics.

[24] Zhang Q., Khan M.U., Lin X., Cai H., Lei H. (2019). Temperature varied biochar as a reinforcing filler for high-density polyethylene composites. Compos. Part B Eng..

[25] Zhang Q., Zhang D., Lu W., Khan M.U., Xu H., Yi W., Lei H., Huo E., Qian M., Zhao Y. (2020). Production of high-density polyethylene biocomposites from rice husk biochar: Effects of varying pyrolysis temperature. Sci. Total Environ..

[26] Dutta B., Raghavan V.G.S., Orsat V., Ngadi M. (2015). Surface characterisation and classification of microwave pyrolysed maple wood biochar. Biosyst. Eng..

[27] Li Y., Liu Y., Liu Y., Chen Y., Chen L., Yan H., Chen Y., Xu F., Li M., Li L. (2022). Modification of sludge biochar by MnO_2_ to degrade methylene blue: Synergistic catalysis and degradation mechanisms. J. Water Process Eng..

[28] Zhou L., Huang Y., Qiu W., Sun Z., Liu Z., Song Z. (2017). Adsorption properties of nano-MnO_2_–biochar composites for copper in aqueous solution. Molecules.

[29] Chen Y.-D., Ho S.-H., Wang D., Wei Z.-S., Chang J.-S., Ren N.-Q. (2018). Lead removal by a magnetic biochar derived from persulfate-ZVI treated sludge together with one-pot pyrolysis. Bioresour. Technol..

[30] Biswas B., Pandey N., Bisht Y., Singh R., Kumar J., Bhaskar T. (2017). Pyrolysis of agricultural biomass residues: Comparative study of corn cob, wheat straw, rice straw and rice husk. Bioresour. Technol..

[31] Siddique M.A.B., Bithi U.H., Ahmed A.N., Gafur M., Reaz A.H., Roy C.K., Islam M.M., Firoz S.H. (2022). Preparation of manganese oxide nanoparticles with enhanced capacitive properties utilizing gel formation method. ACS Omega.

[32] Zhu H., Zou H. (2022). Ultra-efficient catalytic degradation of malachite green dye wastewater by KMnO_4_-modified biochar (Mn/SRBC). RSC Adv..

[33] Li R., Wang Z., Zhao X., Li X., Xie X. (2018). Magnetic biochar-based manganese oxide composite for enhanced fluoroquinolone antibiotic removal from water. Environ. Sci. Pollut. Res..

[34] Zhang M., Lin K., Li X., Wu L., Yu J., Cao S., Zhang D., Xu L., Parikh S.J., Ok Y.S. (2022). Removal of phosphate from water by paper mill sludge biochar. Environ. Pollut..

